# Technical comparison of Abbott’s UroVysion^®^ and Biocare’s CytoFISH urine fluorescence in situ hybridization (FISH) assays

**DOI:** 10.1186/s12935-023-03156-6

**Published:** 2023-12-08

**Authors:** Tammy Anderson, Sharon Hartman, William Dunn, Harvey Bellin, Thomas W. Ehlers, Sarah Groen, Jason A. Ramos

**Affiliations:** 1 Department of Pathology, MidLantic Urology, LLC, 211 South Gulph Road, Suite 211, King of Prussia, PA 19458 USA; 2 Biocare Medical, LLC, Concord, CA USA

**Keywords:** 5p15.2, Bladder cancer, FISH, 9p21, Urinary, Aneuploidy

## Abstract

**Background:**

This study aims to compare the technical performance of Abbott’s UroVysion and Biocare’s CytoFISH urine cytology probe panel and position the CytoFISH probe panel as an alternative to UroVysion. The CytoFISH probe panel was developed based on clinically sensitive chromosomes found to be amplified in bladder cancers, as well as a locus-specific probe also seen to be amplified in bladder tumors. After extensive testing comparing CytoFISH to UroVysion, we present here our findings for the two assays.

**Materials and methods:**

A total of 216 cases representing a mix of male (ages 36–99) and female (ages 46–91) patients were assayed with both probe sets. The CytoFISH and UroVysion probe panels were tested in accordance with the UroVysion procedure, as outlined in the manufacturer’s supplied package insert with the following exception: the probe volume used was 3µL for UroVysion and 5µL for CytoFISH.

**Results:**

The scoring used for the CytoFISH and UroVysion assays revealed a 95% concordance, suggesting that Biocare’s CytoFISH Test has at least the same clinical sensitivity and specificity as claimed by the Abbott UroVysion Kit. We found that the CytoFISH 5p15.2 locus-specific probe was easier to score than UroVysion’s 9p21 deletion.

**Conclusion:**

The high rate of concordance between the two assays suggests that Biocare’s CytoFISH assay is a robust alternative to Abbott’s UroVysion in the diagnosis and monitoring of bladder carcinoma.

## Introduction

Chromosomal aneuploidy, the gain or loss of chromosomes, is the most common type of aberration in cancer cells [1]. The UroVysion® Bladder Cancer Kit (Abbott Laboratories, Abbott Park, IL, USA) was developed based on these principles [2]. The UroVysion Bladder Cancer Kit (UroVysion) is designed to detect aneuploidy for chromosomes 3, 7, 17, and deletion at the locus 9p21, via four-color fluorescence in situ hybridization (FISH) in human urine specimens.

Based on numerous case–control and cohort studies, UroVysion is capable of detecting bladder cancer. The UroVysion assay has high sensitivity (81%) and specificity (96%) for high-grade tumors but lower sensitivity (36–57%) for low-grade tumors [3]. The low sensitivity for the detection of low-grade bladder tumors by UroVysion was one motivation to evaluate additional chromosomal probes in the hopes of developing a more sensitive and specific test for both high- and low-grade bladder tumors.

As an alternative to 9p21, Biocare’s CytoFISH probe panel for urine cytology includes a probe that hybridizes to the locus 5p15.2, for the purposes of detecting amplification events. While loss of signal at 9p21 has generally been regarded as indicative of bladder cancer progression, amplification at 5p15.2 is also strongly associated with disease progression, specifically with high-grade, advanced-stage bladder tumors and rapid tumor cell proliferation in urinary cancer [4]. Cytogenetic analysis also demonstrated that the p arm of Chromosome 5 (5p) might be involved in translocations and/or formation of isochromosomes in a substantial number of bladder tumors [5]. Gains at Chromosomes 3, 7, and 10 are seen more frequently in invasive urothelial tumors, which justifies the usage of these probes as part of the CytoFISH probe offering. This study seeks to technically compare the performance of Biocare’s CytoFISH assay and Abbott’s UroVysion assay, demonstrating the use of both assays as comparable tools for the detection of bladder carcinoma in urine, and positioning CytoFISH as a potential alternative to UroVysion.

## Materials and methods

### Sample collection for CytoFISH and UroVysion

Over a nine-month period, urine specimens (35-60mL) were freshly collected from a mix of male (ages 36–99) and female (ages 46–91) patients. Cases were not consecutive but based on sufficient sample volume for both UroVysion and CytoFISH testing. The specimens were mixed well with fixative at a ratio of 2:1 (urine:fixative), to prevent bacterial growth and preserve cells (Hologic, Inc., Bedford, MA, USA). Urothelial slides were prepared using the Cytyc ThinPrep® 2000 Processor (Hologic, Inc.) following the manufacturer’s instructions, followed by a fixation process using 4:1 (methanol:acetic acid) Carynoy’s solution for 10 min. Both assays were performed in accordance with the UroVysion procedure, as outlined in the manufacturer’s supplied package insert (Abbott Laboratories, Abbott Park, IL, USA) with the following exception: the probe volume used was 3µLfor UroVysion and 5µL for CytoFISH.

Utilizing Abbott’s VP2000 automated pre-hybridization procedure, the slides were partially digested in pepsin (2500–3000 units/mg; Sigma-Aldrich Co. LLC, St. Louis, MO, USA) at 37°C for 10 min. The cells were fixed for 5 min in 1% formaldehyde solution and rinsed in phosphate-buffered saline (PBS) for 5 min. Slides were dehydrated in 70%, 85%, and 100% alcohol for 1 min each and air dried. Slides were hybridized with 5µL of CytoFISH probe mix (COPY CONTROL 3 Aqua FISH Probe/ COPY CONTROL 7 Orange FISH Probe/ COPY CONTROL 10 Green FISH Probe/ 5p15.2 Red FISH Probe; Biocare Medical, LLC, Concord, CA, USA), and 3µL of Abbott’s UroVysion probe mix. Coverslips were applied and sealed with rubber cement, and placed on the ThermoBrite™ Hybridizer (Abbott Laboratories, Abbott Park, IL, USA); denaturing temperature was set to 74 ± 2°C for 3 min, with hybridization temperature set to 39˚C for 16 h. After hybridization, the slides were removed from the ThermoBrite hybridizer. Rubber cement and coverslips were then removed. To remove unbound or nonspecifically hybridized probes, the slides were washed in 0.4X Saline Sodium Citrate (SSC)/0.3% NP-40 for 2 min at 73° ± 1°C, with gentle agitation every 30 s. This was followed by a second wash in 2X SSC/0.1% NP-40 for 1 min, at room temperature, with gentle agitation every 30 s. The slides were placed in a darkened area on a paper towel and allowed to dry completely. Slides were counterstained with 8µL of 4′,6-diamidino-2-phenylindole dihydrochloride (DAPI), coverslipped and then analyzed with an epifluorescence microscope.

### Scoring criteria for CytoFISH Test

Scoring criteria were established following the UroVysion Kit Package Insert (Abbott Laboratories, Abbott Park, IL, USA). The normal cutoff was determined to be three chromosomally abnormal cells maximum and the scoring criteria are:

1. score at minimum 25 cells with abnormal nuclear morphology;
2. a positive cell must show multiple chromosomal gains or amplification of 5p15.2 (Fig. 1);
  **Fig. 1:**
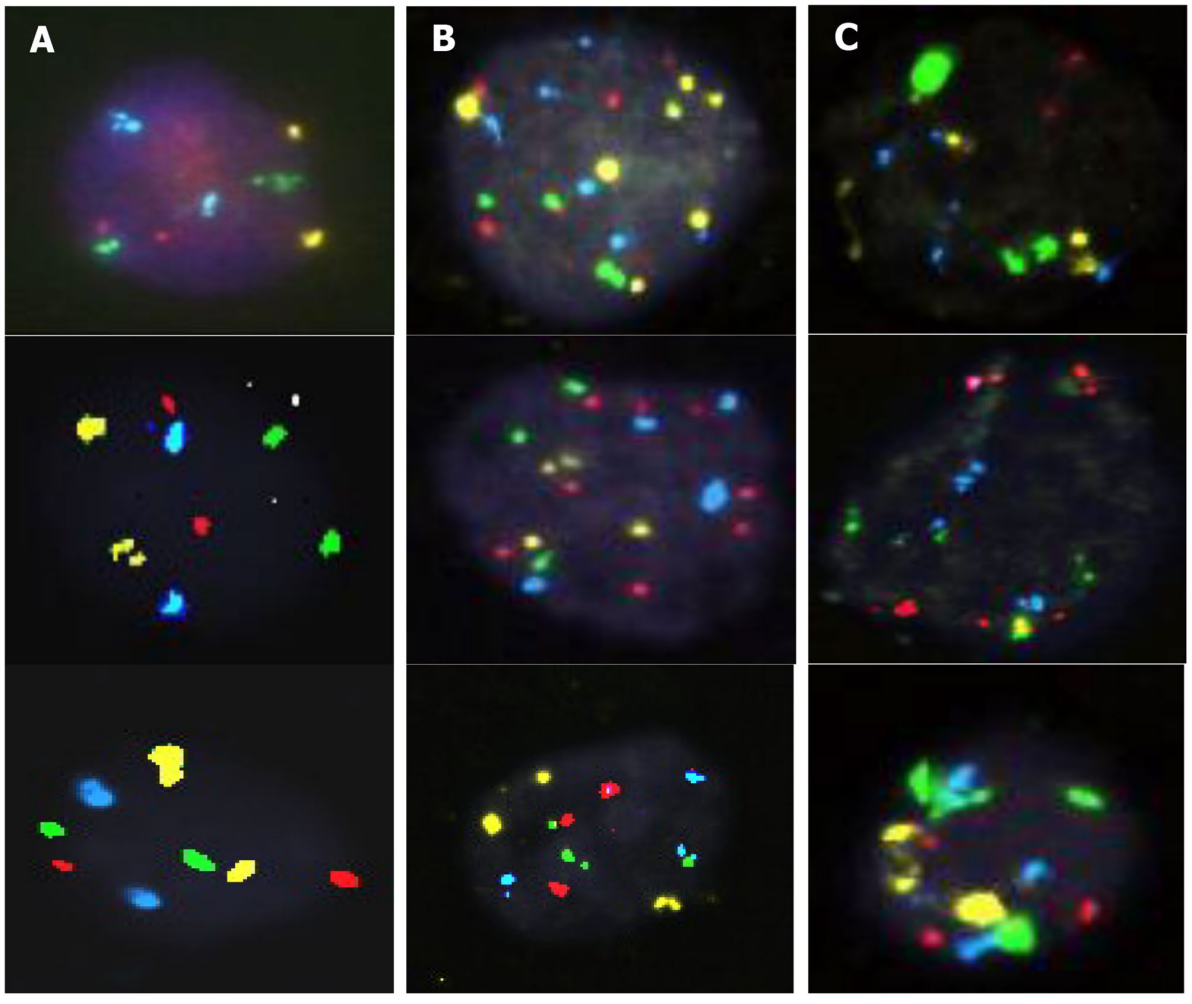
Examples of **(A)** normal urothelial cells, **(B)** urothelial cells with chromosomal gains and 5p15.2 amplifications, and **(C)** suspicious cases
3. if ≥ 4 cells show gains for two or more chromosomes in the same cell, the sample is positive;
4. if < 4 positive cells are found in at least 25 cells, the sample is negative;
5. if three cells with gains of multiple chromosomes are found, analysis is extended until a fourth positive cell is found, or there are no more cells to score;
6. if the sample is negative and < 25 cells can be scored, the analysis is invalid.

### Scoring criteria for UroVysion Kit

Scoring criteria were established following the UroVysion Kit Package Insert (Abbott Laboratories, Abbott Park, IL, USA). The normal cutoff was determined to be three chromosomally abnormal cells maximum and the scoring criteria are:

1. score at minimum 25 cells with abnormal nuclear morphology;
2. a positive cell must show multiple chromosomal gains or loss of both copies of 9p21 locus);
3. if ≥ 4 cells show gains for two or more chromosomes in the same cell, the sample is positive;
4. if ≥ 12 cells have zero 9p21 signals, the sample is positive;
5. if < 4 cells with gains of multiple chromosomes or < 12 cells with homozygous loss of 9p21 are detected in at least 25 cells, the sample is negative;
6. if three cells with gains of multiple chromosomes or 11 cells with homozygous loss of 9p21 are found, analysis is continued until a fourth multiple chromosome positive cell or 9p21 homozygous loss positive cell is found, or there are no more cells to score;
7. if the sample is negative and < 25 cells can be scored, the analysis is invalid.

### Analytical performance of the CytoFISH probe panel

The analytical performance of the CytoFISH panel was validated based on a broadly applicable preclinical process previously described for hybridizing probes [6]. The CytoFISH panel was validated against normal cells in metaphase from five chromosomally normal individuals to determine analytical specificity and sensitivity. Hybridization was limited to the intended target regions of the 4 probes; no cross-hybridization to other chromosome loci was observed in any of the cells examined.

## Results

### Comparison of technical performance of CytoFISH and UroVysion

**Fig. 2:**
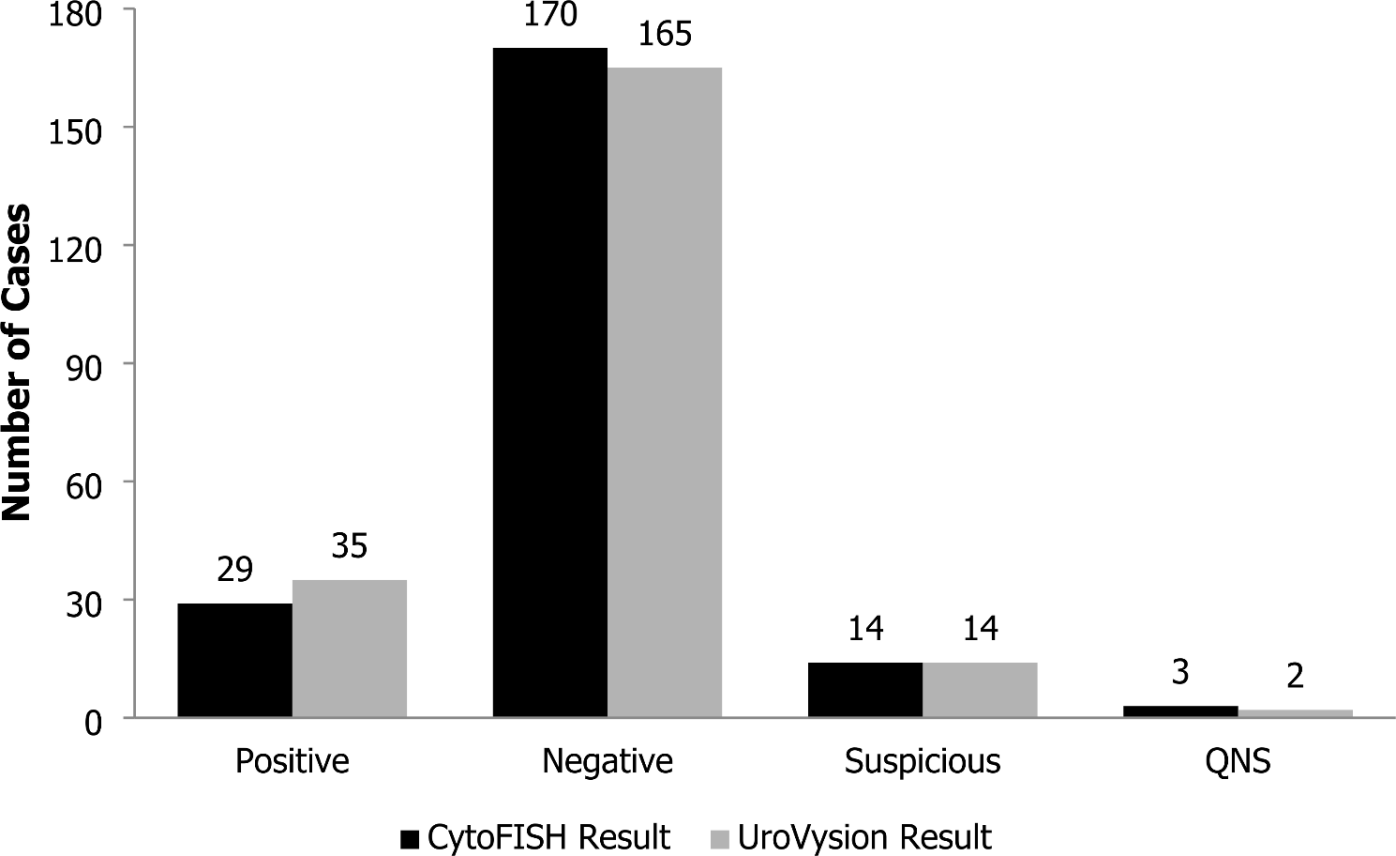
Number of patient cases scored by CytoFISH Test or UroVysion Kit. For CytoFISH, the cases for each scoring category are represented by black bars. For UroVysion, the cases for each scoring category are represented by gray bars

In this study, 216 cases from patients with hematuria suspected of having bladder cancer were analyzed using both the CytoFISH Test and UroVysion Kit (Fig. 2).

Of those 216 cases, 29 were scored positive, 170 were scored negative, 14 were scored suspicious, and 3 samples did not have sufficient quantity of cells to score (QNS) using the CytoFISH Test. For the UroVysion Kit, out of the 216 cases, 35 cases were scored positive, 165 were scored negative, 14 were scored suspicious, and 2 were QNS.

To determine concordance between CytoFISH and UroVysion, individual case results were compared between the two assays (Table 1).

**Table 1:** Concordance between CytoFISH and UroVysion

	CytoFISH Result	UroVysion Result	Concordant Cases
**Positive**	29	35	29
**Negative**	170	165	163
**Suspicious**	14	14	14
**QNS**	3	2	N/A
**Total**	**216**	**216**	**206**

In this comparison, 29 were scored positive in both CytoFISH and UroVysion. 163 cases were scored as negative in both CytoFISH and UroVysion. While the scoring criteria laid out above are delineated positive/negative, there were 14 cases which fell into the third category labeled as suspicious for both the CytoFISH Test and UroVysion Kit. These 14 suspicious cases demonstrated the clear presence of abnormal cells; however, there were too few present to count as truly positive. The total of 206 concordant cases represents a 95% concordance between CytoFISH and UroVysion, suggesting that Biocare’s CytoFISH Test has at least the same clinical sensitivity and specificity as claimed by the Abbott UroVysion Kit.

## Discussion

Chromosomal aneuploidy has been documented as a hallmark of cancer, with recent studies focusing on the origin of these aberrations and how they profile cancer genomes [6, 7]. Chromosomes 3, 7, and 10 have been shown to be amplified in urothelial tumors, making these chromosomes prime targets for FISH aneuploidy analyses for bladder cancer detection [8]. Another such target is the 5p15.2 locus. 5p15.2 amplification has shown strong association with rapid tumor cell proliferation in urinary cancer [4]. The addition of a locus-specific probe to the CytoFISH panel was by design. Chromosome-enumeration probes are effective tools that make it possible to detect numerical chromosome imbalances (such as aneuploidy) in cell populations. The number of signals for these probes should match numbers of homologous chromosomes per interphase nucleus. However, that is not always the case [9, 10]. As a result, there may be limitations on their use. Locus-specific probes successfully overcome such limitations, giving more precise copy number information [11]. The CytoFISH panel was developed to detect aneuploidy for chromosomes 3, 7, and 10, as well as 5p15.2 locus amplification.

The UroVysion Kit was developed over 10 years ago to overcome the sensitivity shortcomings of urine cytology. During this time, UroVysion has been shown to have high sensitivity in certain clinical situations, but has also been shown to have lower specificity when compared to cytology [12]. The UroVysion Kit detects and quantifies chromosomes 3, 7, and 17 and the locus 9p21 on urothelial slides obtained from urine using four colors of fluorescently labeled DNA probes visualized with an epifluorescence microscope [3].

One of the challenges of using 9p21 is the distinction between a true loss of signal and weak detection of it. So, it became worthwhile to look at amplification as a potential alternative companion to chromosomal proliferation and a determinant of disease progression. The performance for the CytoFISH bladder cancer probe panel and UroVysion compared quite favorably. The rate of concordance (206 out of the 216 cases compared; 95%) is high, and most likely would have reached 100% had enough sample in the QNS cases been available, as clear results were obtained for at least one of the assays in each of those instances. The goal of this study was to show equivalent performance between UroVysion and CytoFISH, which has been achieved. Additional studies, with a larger patient cohort, are needed to assess the clinical sensitivity and specificity of CytoFISH compared to standard of care (cystoscopy/histology).

As for the 6 cases of discordance seen in the comparison (Table 1, positive results), there were technical/environmental issues with sampling. Sample collection, preservation, and quality can be recurrently challenged by the effect of winter in the region, which, in turn, affects the performance of both assays. Coincident with seasonal conditions of low temperature and humidity, and icy or wet conditions, sample quality can be compromised by cellular debris, white blood cell count, bacteria, fungi, ice crystals, and other seasonal factors that lead to declined assay performance. The decline is characterized by weak probe signals and high background noise. During the sample collection for those cases, there was a spike of particularly cold weather that contributed to the discrepancy mentioned above. Once the cold weather subsided, both assays recovered their performance. This alleviated stress on sample collection, restoring the concordance rate to near 100%.

## Conclusion

This study has sought to compare the performance of a non-predictive value assay to Abbott’s FDA-approved method for urine FISH applications. The motivation to do so is derived from looking beyond chromosomal proliferation to examine a locus-specific event other than deletion, which could potentially benefit diagnosis of patients suspected of having urothelial carcinoma. Performing the CytoFISH test protocol was similar to that of UroVysion, but the scoring for CytoFISH was simpler due to the fact that each of the targets being visualized were amplification targets. Despite the strong rationale for monitoring the absence of the 9p21 locus as a necessary step for aneuploid cells to continue dividing, the homozygous deletion of this gene is not a very common observation in urine cytology samples. Furthermore, gene *p16* at the 9p21 locus has been shown to be silenced not only by deletion, but also by methylation in bladder cancer [13]. This would render the use of a FISH probe clinically irrelevant in monitoring this gene’s function, as FISH technology cannot determine methylation status [14]. Alternatively, the locus 5p15 offers an opportunity to examine the amplification of several oncogenes, one of which, TRIO, has already been implicated in bladder cancer progression [4]. Given these distinctions, the CytoFISH panel of probes provided an opportunity to assess the amplification events of 5p15.2, and its potential as a diagnostic tool. Based on the high rate of concordance (95%) obtained in this performance assessment, Biocare’s CytoFISH assay appears to be a reliable tool to both diagnose and monitor bladder cancer. Future directions of this work will examine in more depth how amplification at 5p15.2 complements chromosomal proliferation in disease diagnosis.

## Data Availability

All data supporting this study is maintained. Please send all data requests to sgroen@biocare.net.
